# The Relationship between Statin and Risk of Age-Related Macular Degeneration: A Systematic Review and Meta-Analysis

**DOI:** 10.1155/2022/8564818

**Published:** 2022-05-09

**Authors:** Ezatollah Memarzadeh, Saeid Heidari-Soureshjani

**Affiliations:** ^1^Department of Ophthalmology, School of Medicine, Kashani Hospital, Shahrekord University of Medical Sciences, Shahrekord, Iran; ^2^Modeling in Health Research Center, Shahrekord University of Medical Sciences, Shahrekord, Iran

## Abstract

**Methods:**

Web of Science, PubMed, and Scopus databases were searched for articles that addressed the relationship between statin consumption and risk of AMD. The pooled odds ratio (OR) and 95% confidence interval (CI) were calculated using a random-effects model. Subgroup analyses and sensitivity analyses were also conducted. Cochran's *Q* test and the I^2^ statistic were used to evaluate the heterogeneity. To assess potential publication bias, Begg's test was used.

**Results:**

In total, 22 studies were reviewed in the meta-analysis that included 2063195 participants and 313702 (15.20%) AMD patients compared to individuals not receiving statins. The OR of AMD in statin-receiving participants was 0.93 (95% CI; 0.83–1.05, *P*=0.225). The OR of AMD in those that received statins was 0.92 (95% CI; 0.75–1.13, *P*=0.440) in case-control studies, 0.95 (95% CI; 0.82–1.09, *P*=0.458) in cohort studies, 0.951 (95% CI; 0.59–1.53, *P*=0.831) in cross-sectional studies, 0.94 (95% CI; 0.80–1.10, *P*=0.468) in North America, 0.81 (95% CI; 0.54–1.21, *P*=0.308) in Europe, 1.05 (95% CI; 0.94–1.18, *P*=0.362) in Asia, and 0.52 (95% CI; 0.26–1.04, *P*=0.125) in Australia. No publication bias was observed in this study (*P*=0.114).

**Conclusion:**

According to the results of this study, taking statins does not increase or decrease the risk of AMD development. Therefore, this drug group cannot be considered a protective or risk factor for the occurrence of AMD.

## 1. Introduction

Age-related macular degeneration (AMD) is one of the leading causes of visual disability globally, particularly in people over 60 years. This disorder is responsible for 8.7% of all types of blindness across the world [1]. Vision loss associated with AMD can induce negative effects on the independence of the elderly and their mental health [2]. Advanced AMD leads to bilateral blindness, imposes stupendous healthcare costs, and reduces the patients' quality of life as a serious systemic disease [1–3]. AMD is associated with a disorder in the central area of the retina called the macula that, in developing form, can lead to bilateral ocular problems in senile people. Only 4% of the retinal area is compromised with the macula, but it supports most of the useful photopic human vision. Because of the presence of cone photoreceptor cells at the center of the macula, which is called the fovea, any lesion in this area can lead to ocular impairment [2].

Given the incomplete understanding of the mechanism confounded by delivering the drugs to the posterior segment of the eyeball and unclear AMD molecular pathogenesis, AMD treatment strategies have not been very successful [2, 4]. Inflammation is considered a key element in the pathogenesis of this disease. Therefore, the anti-inflammatory properties of statins in the treatment of AMD have been considered by researchers [5]. Statins, commonly known as hydroxymethylglutaryl coenzyme A (HMG-CoA) reductase inhibitors, are a group of cholesterol-lowering drugs that have been reported as promising agents for preventing AMD [6, 7]. Several meta-analyses were performed at the time of this study. For example, Ma et al. in their study reported that statin consumption has provided a protective effect on early and exudative AMD [8]. In a meta-analysis which was conducted in 2014, there was no association between statin use and the development and progression of AMD [9]. Another meta-analysis demonstrated that statins do not appear to have a preventative effect on AMD and reduce its risk [10]. However, due to newer cohort and cross-sectional studies in this regard, it was necessary to conduct another meta-analysis study. Given that these conflicting results were related to the impact of statins on AMD prevention, progression, and development and given the need for more effective treatment strategies, we performed this meta-analysis to investigate the relationship between statin consumption and the risk of developing AMD.

## 2. Materials and Methods

### 2.1. Data Sources and Search Strategy

This meta-analysis was performed by following PRISMA guidelines (http://prisma-statement.org/prismastatement/Checklist.aspx). For this purpose, an extensive systematic review was undertaken on 31 January 2021 in PubMed, Web of Science (ISI), and Scopus databases. The following main and MeSH keywords were used to search: ((“Age-related macular degeneration” OR “Age-Related Maculopathies” OR “Age-Related Maculopathy” OR “Macular Degeneration” OR AMD) AND (statin^*∗*^ OR “hydroxymethylglutaryl-CoA reductase” OR “HMGCoA reductase” OR “anticholesteremic” OR “simvastatin” OR “rosuvastatin” OR “pravastatin” OR “atorvastatin” OR “fluvastatin” OR “cerivastatin” OR “pitavastatin” OR “lovastatin”)).

### 2.2. Study Selection

**Figure 1:**
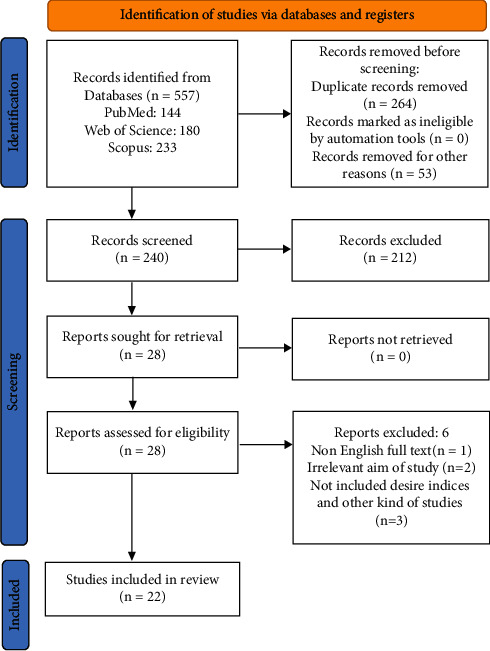
Flowchart for including studies in the meta-analysis.

The peer-reviewed publications were imported into EndNote X8 (8 November 2016, Thomson Reuters) to detect and remove duplicate publications. Two investigators independently screened the titles and abstracts of the studies based on the inclusion and exclusion criteria. In this systematic review and meta-analysis study, studies with cohort design, case-control, cross-sectional, and randomized clinical trials were considered. Based on our inclusion criteria, the studies that addressed the association between statin consumption and the risk of developing AMD subtypes were included in the systematic review and meta-analysis. Also, studies that reported the relative risk (RR) or the odds ratio (OR) of AMD about statin use, or that these indicators could be calculated based on the information presented in the article, were included in our study. The exclusion criteria were lack of access to the full text of the publication and studies published in non-English language. The full texts of all eligible publications were independently reviewed. If any potential disagreement was detected during the review, a consensus was achieved by discussing the disagreement in question with other team members. A flowchart of the search strategy is illustrated in Figure 1.

### 2.3. Data Extraction and Quality Assessment

Data were extracted independently by two individuals, and inconsistencies were resolved through discussion. From the studies included, the following information was extracted: the first author's name, year of publication, country of study, sample size, the mean age of the study population, the length of follow-up, and statistical information including odds ratio (OR) or risk ratio (RR) or hazard ratio (HR) with 95% confidence interval (CI) for AMD patients receiving drugs of the statin group compared to those who did not receive such drugs.

### 2.4. Evaluating the Quality of the Studies

The quality of included observational studies was determined by using the Newcastle–Ottawa scale (NOS) [11] based on selection, comparability, and exposure/outcome. In this systematic review and meta-analysis, studies with a score of at least 7 were considered as being of high quality.

### 2.5. Statistical Analysis

For the meta-analysis, the OR was used to assess the relationship between statin consumption and the risk of developing AMD. The effect size of the relationship between study exposure and the outcome was reported by OR with 95% CI. When the incidence of the disease is low, the odds ratio can be considered an appropriate estimate of the risk ratio. Therefore, in this study, since the incidence of the desired outcome is not high, the odds ratio is used as an estimate of the risk ratio. Random-effects models of the meta-analysis were used to calculate the overall summary estimates. Graphically, the illustration of the individual OR and summary estimates was done using forest plots. Based on *a priori* decisions, subgroup analyses were conducted according to the geographical location (Europe, America, Asia, and Australia), the sample size of the study (˃10,000 vs. ≤10,000), type of the study (case-control, cross-sectional, and cohort), study period (2000–2010 and 2011–2021), and the length of follow-up (>five years or ≤five years).

Heterogeneity among studies was tested by Cochran's *Q* test (reported by the chi-square test and *P* < 0.1 considered as a significance level) and the I^2^ statistic. To clarify the sources of statistical heterogeneity between studies further and evaluate the robustness of the findings, a series of sensitivity analyses were performed. First, we aimed to examine the effect of individual studies on the summary estimates, for which sensitivity analyses were conducted where the pooled estimates were recalculated after deleting one study at each run. Secondly, a meta-regression analysis was conducted to identify the source of differences between studies in the observed effect size.

Potential publication bias and accumulation bias were investigated using Begg's test. All statistical analyses were performed using Stata 14.0 (Stata LLC, College Station, TX, USA). *P* < 0.05 was considered a statistically significant level.

## 3. Results

### 3.1. Search Results and Study Characteristics of Selected Studies

The PRISMA flowchart of the search strategy is illustrated in Figure 1. The initial electronic search retrieved 557 titles/abstracts. From the total articles retrieved, 264 articles were removed due to duplicate titles. Some other titles/abstracts were also excluded (*n* = 6), one because of not having been published in English [12], two because of not fulfilling our aims and criteria [7, 13], three because of not accessing the full text [14–16], and 3 because of being commentary and letters [17–19].

Finally, 22 articles were selected for the final assessment of the association between statin consumption and the risk of developing AMD [6, 20–40].

### 3.2. Characteristics of Selected Studies regarding the Association between the Statin Group Receiving Drugs and the Risk of AMD

A total of 2063195 participants and 313702 (15.20%) AMD patients were included in the reviewed articles. Among the studies, 11 used a cohort design with a sample size of 1462588 participants and 255970 (17.50%) AMD patients [20–30], eight had a case-control design with a sample size of 588648 participants and 54776 (9.30%) AMD patients [6, 31–37], and three had a cross-sectional design with a sample size of 11959 participants and 2956 (24.71%) AMD patients [38–40] (Table 1).

The articles included in this systematic review and meta-analysis were published between 2003 and 2021. The studies had been conducted in diverse geographical regions, with thirteen conducted in North America [6, 20, 21, 23, 26, 28–30, 33, 34, 36, 39, 40], four in Europe [24, 27, 31, 32], four in Asia [25, 35, 37, 38], and one in Australia [22]. The mean age of the participants in the studies was 67.91 years (Table 1).

In each of the studies included in the meta-analysis, to estimate the effect of statin consumption on AMD development, the role of several variables was adjusted as confounding variables using statistical techniques.  Table 2 shows the variables that were adjusted in each study.

### 3.3. Statin Consumption and Risk of Developing AMD

**Figure 2:**
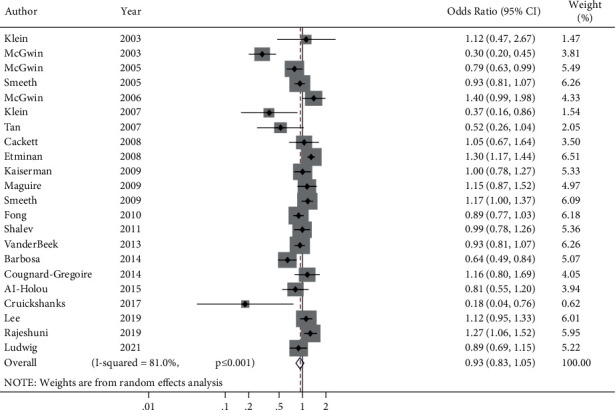
The forest plot of the relationship between statin consumption and risk of developing age-related macular degeneration.

Compared to people who did not receive statins, the OR of AMD development in participants who received statins was 0.93 (95% CI; 0.83–1.05, *P*=0.225). The OR for the association between statin consumption and risk of developing any AMD is illustrated in Figure 2.

There was significant heterogeneity in the results in the meta-analysis (chi-square = 110.521 (df = 21), *P* ≤ 0.001, and I^2^ = 81%) and between-study variance (tau squared = 0.0535). To investigate the cause of heterogeneity in the results of studies, a meta-regression was performed in which the following variables were included: year, follow-up term, study type/design, sample size, quality of the study based on the Newcastle–Ottawa Scale, study period, and geographical locations. The results from the meta-regression analysis determined that there was no significant source of heterogeneity (*P* > 0.10).

**Figure 3:**
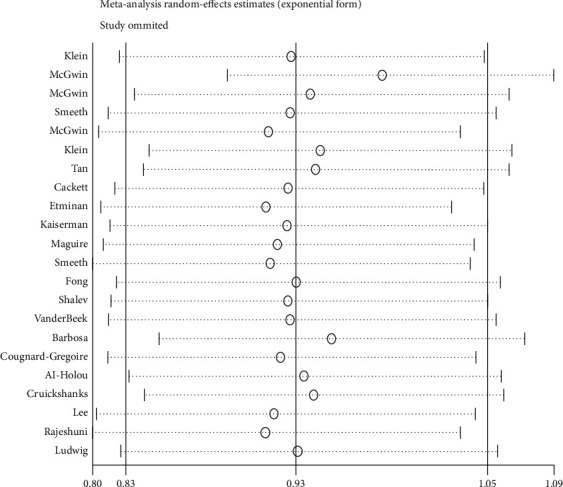
Sensitivity analysis for the assessment of the relationship between statin consumption and risk of developing AMD.

Moreover, sensitivity analysis was performed by excluding studies from the analysis one by one at each run. However, the estimated OR did not change significantly, further indicating the robustness of the results (Table 3 and Figure 3).

### 3.4. Subgroup Analysis

Subgroup analysis was performed to investigate the association between statin consumption and risk of developing AMD based on study design, sample size, length of the follow-up period, study period, and geographical location. The OR of AMD in individuals who received statins was 0.92 (95% CI; 0.75–1.13, *P*=0.440) in case-control studies, 0.95 (95% CI; 0.82–1.09, *P*=0.458) in cohort studies, and 0.951(95% CI; 0.59–1.53, *P*=0.831) in cross-sectional studies (Table 4). Regarding geographical locations, the OR of AMD was 0.94 (95% CI; 0.80–1.10, *P*=0.468) in North America, 0.81 (95% CI; 0.54–1.21, *P*=0.308) in Europe, 1.05 (95% CI; 0.94–1.18, *P*=0.362) in Asia, and 0.52 (95% CI; 0.26–1.04, *P*=0.125) in Australia (Table 4). As seen in Table 4, the OR (95% CI) of the relationship between statin consumption and AMD development was not statistically significant when subgroup analysis was performed for the study period, sample size, and length of the follow-up period.

### 3.5. Evaluation of Publication Bias Related to Statin Consumption and Risk of Developing AMD

**Figure 4:**
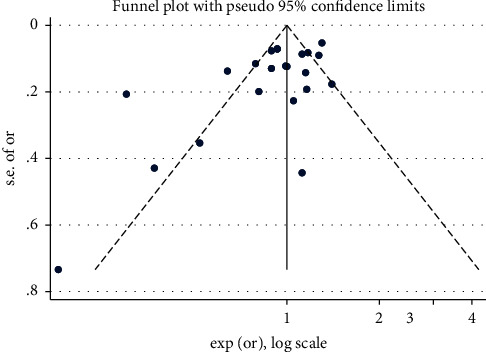
The funnel plot of the relationship between statin consumption and risk of AMD.

Regarding the relationship between statin consumption and developing AMD, Begg's test results (*P*=0.114) showed no publication bias (Figure 4).

## 4. Discussion

Age-related macular degeneration (AMD) is one of the leading causes of central vision loss in people aged 65 years and older. It seems that an important risk factor for this disease is high fat intake in diets. It can be an important factor in the development and progression of vascular and retinal disease. Therefore, it can be assumed that high cholesterol and AMD development are related [41, 42]. Therefore, taking cholesterol-lowering medications, such as statins, may be able to prevent or delay the onset and progression of AMD [29, 30, 40]. However, the results and findings of studies in this field are not conclusive and transparent [28–30, 39, 40]. Consequently, given the importance of the issue and the potential inhibitory effects of statins on the onset and development of AMD, it is important to continuously review and evaluate emerging findings related to this question. This meta-analysis aimed at investigating the association between statin consumption and the risk of developing AMD. Our study showed that statins had no protective or risk effect on AMD development, so the OR of AMD development in participants who received statins was 0.93 (95% CI; 0.83–1.05, *P*=0.225).

In a study conducted by Chuo et al. in 2007 entitled “Use of Lipid-Lowering Agents for the Prevention of Age-Related Macular Degeneration: A Meta-Analysis of Observational Studies,” there was no statistically significant relationship between receiving lipid-lowering agents and the development of age-related macular degeneration (AMD), so the pooled relative risk (RR) of the development of age-related macular degeneration (AMD) in users compared to nonusers of lipid-lowering drugs was equal to 0.74 (95% CI, 0.55–1.00). Similarly, when only studies were considered in the analysis that used the statins as a lipid-lowering regimen, it was observed that the relative risk of development of AMD was equal to 0.70 (95% CI, 0.48–1.03). Therefore, in this study, it was concluded that receiving fat-reducing agents, such as statins, do not significantly reduce the risk of AMD [10].

In another systematic review and meta-analysis study conducted by Ma et al. in 2016, which evaluated the association between statin use and the risk of age-related macular degeneration (AMD), also no significant association was observed between statin use and the risk of any AMD (RR, 0.95; 95% CI, 0.74–1.15). However, in the stratified analysis, the results of the study showed a significant protective effect of statins in the early stages of AMD so that the relative risk of early stages of AMD was equal to 0.83 (95% CI, 0.66–0.99). Nevertheless, the association between statins and late age-related macular degeneration was not significant, so the relative risk of late age-related macular degeneration was equal to 0.92 (95% CI, 0.77–1.07) [8]. Similarly, in another meta-analysis conducted by Klein et al., results demonstrated that there was no association between statin use and the development and progression of AMD. In this study, after multiple testing corrections, there was no statistically significant relationship between statin use or serum lipid genes and any subtypes of the AMD outcomes [9]. So, unlike some research that shows protective effects [29, 31] or risk factor effects [34, 40] of statins against AMD development, so far, based on the results of meta-analysis studies in this field, no statistically significant relationship has been observed. Therefore, to achieve a clear and concise result, it is still necessary to conduct large studies with appropriate design, especially studies with a randomized clinical trial structure.

This systematic review and meta-analysis study was performed by conducting a comprehensive search for the studies related to AMD. Statistical tests were used to estimate the effect size of missing studies, perform sensitivity analysis, and assess the influence of subgroups on the strengths of the reviewed article. However, restricting the search to English-language articles has led to language bias in this article. In addition, although for estimation of the effect of statins on AMD development, adjusted effect sizes have been considered in each study, it should be noted that in each study, different variables have been considered for adjustment, so a meta-analysis on the results of these studies is problematic because the effect size reported in each study has not been matched to a complete set of confounder variables. Therefore, to obtain clear results regarding statins and AMD development and progress, it is necessary to conduct randomized clinical trial studies or large cohort studies that have appropriately controlled the role of confounder variables and prevented the occurrence of common biases in the study. Also, the criteria used to define and grade AMD are very different between studies conducted in different countries and periods, which can be a reason for the high heterogeneity observed between the results of studies entered in the meta-analysis. Publication bias is also one of the concerns about the results of this study, although the results of the statistical test used in this regard were not significant; however, it is difficult to completely rule out the existence of publication bias and its effect on the final results of the study.

## 5. Conclusion

According to the results of this study, taking statins does not increase or decrease the risk of AMD development. Therefore, this drug group cannot be considered a protective or risk factor for the occurrence of AMD. However, before drawing definitive conclusions, randomized clinical trials or cohort studies with large sample sizes are required to investigate the relationship between receiving statins and AMD.

## Figures and Tables

**Table 1:** Characteristics of studies reviewed for the relationship between statin consumption and risk of developing age-related macular degeneration.

Publication lead author	Year	Study setting	Study design	Sample size	No. of hip fractures	Age, mean (years)	OR	95% CI	Follow-up (months)	NOS
McGwin [31]	2003	UK	Case-control	6,050	550	73.05	0.3	(0.2–0.45)	4	7
Klein [20]	2003	USA	Cohort	3,684	118	63.8	1.12	(0.47–2.67)	5	7
Smeeth [32]	2005	UK	Case-control	102,383	17,632	77.4	0.93	(0.81–1.07)	5	9
McGwin [6]	2005	USA	Case-control	12,588	871	60.9	0.79	(0.63–0.99)	6	7
McGwin [33]	2006	USA	Case-control	2,755	390	78.3	1.4	(0.99–1.98)	3	7
Tan [22]	2007	Australia	Cohort	2,335	2,335	64.4	0.52	(0.26–1.04)	10	7
Klein [21]	2007	USA	Cohort	6,176	919	63.9	0.37	(0.16–0.86)	2	7
Cackett [38]	2008	Singapore	Cross-sectional	3,265	190	57.45	1.05	(0.67–1.63)	3	6
Etminan [34]	2008	Canada	Case-control	14,335	2,867	70.2	1.3	(0.17–1.44)	7	6
Kaiserman [35]	2009	Israel	Case-control	139,894	29,417	66.09	1	(0.80–1.30)	3	7
Maguire [23]	2009	USA	Cohort	744	296	70	1.15	(0.87–1.52)	2	7
Smeeth [24]	2009	UK	Cohort	613,229	12,988	72.1	1.17	(1–1.38)	4.4	9
Fong [36]	2010	USA	Case-control	79,369	719	75.65	0.89	(0.77–1.04)	5	7
Shalev [25]	2011	Israel	Cohort	108,973	2,732	65.55	0.99	(0.78–1.26)	5	7
VanderBeek [26]	2013	USA	Cohort	486,124	223,104	68.2	0.93	(0.81–1.07)	2	7
Cougnard-Gregoire [27]	2014	France	Cohort	825	285	80.2	1.16	(0.80–1.7)	8	8
Barbosa [39]	2014	USA	Cross-sectional	5,604	1,231	61.8	0.64	(0.49–0.84)	3	8
Al-Holou [28]	2015	USA	Cohort	3,791	1,659	73.21	0.81	(0.55–1.2)	2	6
Cruickshanks [29]	2017	USA	Cohort	4,819	204	54	0.18	(0.04–0.71)	5	9
Lee [37]	2019	South Korea	Case-control	231,274	2,330	66.45	1.12	(0.94–1.32)	13	7
Rajeshuni [40]	2019	USA	Cross-sectional	3,090	1,535	85	1.27	(1.06–1.51)	1	6
Ludwig [30]	2021	USA	Cohort	231,888	11,330	46.5	0.89	(0.83–1.38)	8	7

**Table 2:** Adjusted variables in the assessment relationship between statin consumption and the risk of developing age-related macular degeneration.

First author	Year	Adjusted variables
McGwin [31]	2003	Diabetes, lipid metabolism disorders, hypertension, ischemic heart disease, cerebrovascular disease, and arterial disease
Klein [20]	2003	Age and sex
Smeeth [32]	2005	Consultation rate, smoking, alcohol intake, BMI, atherosclerosis, hyperlipidemia, heart failure, diabetes, hypertension, cardiovascular drug use, and fibrate use
McGwin [6]	2005	Age, gender, and race
McGwin [33]	2006	Age, race, and sex
Tan [22]	2007	Age, gender, smoking, job prestige, history of cardiovascular disease (angina, myocardial infarction, or stroke), white cell count, fibrinogen, and total high-density lipoprotein cholesterol
Klein [21]	2007	Age, sex, race, and study site
Cackett [38]	2008	Age, gender, smoking status, hypertension, and history of myocardial infarction
Etminan [34]	2008	Age, gender, comorbidity, prior history of diabetic medications, myocardial infarction, stroke, ischemic heart disease, and congestive heart disease
Kaiserman [35]	2009	Age, gender, socioeconomic status, place of birth, place of residence, hyperlipidemia, hypertension, ischemic heart disease, diabetes, and congestive heart failure
Maguire [23]	2009	Age, gender, race, smoking, and hypertension
Smeeth [24]	2009	Age, sex, propensity score, year of the index, the first diagnosis of any of the following postindex dates: Diabetes, cerebrovascular disease
Fong [36]	2010	Age, gender, and history of myocardial infarction or stroke
Shalev [25]	2011	Age, socioeconomic level, no. of ophthalmologist visits, no. of GP visits, and chronic conditions
VanderBeek [26]	2013	Sociodemographic factors (sex, race, education level, and household net worth), comorbid ocular conditions (cataract, pseudophakia/aphakia, open-angle glaucoma, and diabetic retinopathy), and systemic medical conditions
Cougnard-Gregoire [27]	2014	Age, gender, educational level, smoking, BMI, hypertension, HDL, LDL, triglycerides, cardiovascular disease, diabetes, ApoE2, ApoE4, CFH Y402H, ARMS2 A69S, LIPC (rs10468017), LIPC(rs493258), LPL, and ABCA
Barbosa [39]	2014	Age, gender, other demographic characteristics, health-related behaviors, comorbidities, and self-reported general health condition
Al-Holou [28]	2015	Age, gender, smoking status, aspirin use, history of diabetes, hypertension, heart disease, angina, and stroke
Cruickshanks [29]	2017	Age, sex, smoking, educational attainment, exercise, levels of non-high-density lipoprotein cholesterol and high-sensitivity C-reactive protein, and use of nonsteroidal anti-inflammatory drugs, statins, and multivitamins
Lee [37]	2019	Socioeconomic status, healthcare resource utilization, combined medication use, and comorbidities
Rajeshuni [40]	2019	Age, sex, race, and comorbidity status
Ludwig [30]	2021	Age at index diagnosis of nonexudative AMD, sex, smoking, comorbidities from the elixhauser comorbidity index, and use of anticoagulants, antihypertensives, or diuretics

**Table 3:** Sensitivity analysis for the assessment of the relationship between statin consumption and risk of developing AMD.

First author	Year	OR (95% CI)	*p* value
Klein [20]	2003	0.92 (0.82–1.04)	0.125
McGwin [31]	2003	0.98 (0.88–1.08)	0.314
McGwin [6]	2005	0.94 (0.83–1.05)	0.227
Smeeth [32]	2005	0.92 (0.81–1.05)	0.211
McGwin [33]	2006	0.91 (0.80–1.02)	0.116
Klein [21]	2007	0.94 (0.84–1.06)	0.249
Tan [22]	2007	0.94 (0.83–1.05)	0.266
Cackett [38]	2008	0.92 (0.82–1.04)	0.124
Etminan [34]	2008	0.91 (0.80–1.02)	0.091
Kaiserman [35]	2009	0.92 (0.81–1.04)	0.115
Maguire [23]	2009	0.91 (0.81–1.04)	0.136
Smeeth [24]	2009	0.91 (0.80–1.03)	0.154
Fong [36]	2010	0.92 (0.82–1.05)	0.209
Shalev [25]	2011	0.92 (0.81–1.04)	0.115
VanderBeek [26]	2013	0.92 (0.81–1.05)	0.135
Barbosa [39]	2014	0.95 (0.84–1.07)	0.284
Cougnard-Gregoire [27]	2014	0.92 (0.81–1.04)	0.109
Al-Holou [28]	2015	0.93 (0.83–1.05)	0.275
Cruickshanks [29]	2017	0.94 (0.84–1.06)	0.244
Lee [37]	2019	0.91 (0.80–1.04)	0.322
Rajeshuni [40]	2019	0.91 (0.80–1.03)	0.165
Ludwig [30]	2021	0.93 (0.82–1.05)	0.205

**Table 4:** Subgroup analysis of the relationship between statin consumption and risk of developing age-related macular degeneration.

Characteristics		Study no.	OR (95% CI)	*p* value
Study type	Cross-sectional	2	0.95 (0.59–1.53)	0.831
	Cohort	13	0.95 (0.82–1.09)	0.458
	Case-control	8	0.92 (0.75–1.13)	0.440

Study location	North America and Canada	13	0.94 (0.80–1.10)	0.468
	Europe	4	0.81 (0.54–1.21)	0.308
	Asia	4	1.05 (0.94–1.18)	0.362
	Australia	1	0.52 (0.26–1.04)	0.125

Study period	2000–2010	13	0.91 (0.76–1.08)	0.291
	2011–2021	9	0.95 (0.81–1.11)	0.520

Follow-up period	Less than five years	16	0.90 (0.78–1.04)	0.165
	More than five years	6	0.99 (0.81–1.22)	0.965

Sample size	>10000	10	1.02 (0.90–1.12)	0.983
	≤10000	12	0.79 (0.59–1.06)	0.114

## References

[1] Stahl A. (2020). The diagnosis and treatment of age-related macular degeneration. *Deutsches Ärzteblatt International*.

[2] Yuzawa M., Fujita K., Tanaka E., Wang C., Wang Y. (2013). Assessing quality of life in the treatment of patients with age-related macular degeneration: clinical research findings and recommendations for clinical practice. *Clinical Ophthalmology*.

[3] Ruiz-Moreno J. M., Arias L., Abraldes M. J., Montero J., Udaondo P., RAMDEBURS study group (2021). Economic burden of age-related macular degeneration in routine clinical practice: the RAMDEBURS study. *International Ophthalmology*.

[4] Wei C. X., Sun A., Yu Y. (2016). Challenges in the development of therapy for dry age-related macular degeneration. *Retinal Degenerative Diseases*.

[5] Guymer R. H., Chiu A. W.-i., Lim L., Baird P. N. (2005). HMG CoA reductase inhibitors (statins): do they have a role in age-related macular degeneration?. *Survey of Ophthalmology*.

[6] McGwin G., Xie A., Owsley C. (2005). The use of cholesterol-lowering medications and age-related macular degeneration. *Ophthalmology*.

[7] Vavvas D. G., Daniels A. B., Kapsala Z. G. (2016). Regression of some high-risk features of age-related macular degeneration (AMD) in patients receiving intensive statin treatment. *EBioMedicine*.

[8] Ma L., Wang Y., Du J., Wang M., Zhang R., Fu Y. (2015). The association between statin use and risk of age-related macular degeneration. *Scientific Reports*.

[9] Klein R., Myers C. E., Buitendijk G. H. S. (2014). Lipids, lipid genes, and incident age-related macular degeneration: the three continent age-related macular degeneration consortium. *American Journal of Ophthalmology*.

[10] Chuo J. Y., Wiens M., Etminan M., Maberley D. A. L. (2007). Use of lipid-lowering agents for the prevention of age-related macular degeneration: a meta-analysis of observational studies. *Ophthalmic Epidemiology*.

[11] Peterson J., Welch V., Losos M., Tugwell P. (2011). *The Newcastle-Ottawa Scale (NOS) for Assessing the Quality of Nonrandomised Studies in Meta-Analyses*.

[12] Martini E., Scorolli L., Burgagni M. S., Fessehaie S. (1991). Valutazione degli effetti retinici della somministrazione di simvastatina in pazienti affetti da degenerazione maculare senile. *Ann Ottalmol Clin Ocul*.

[13] Kananen F., Strandberg T., Loukovaara S. (2021). Early middle age cholesterol levels and the association with age-related macular degeneration. *Acta Ophthalmology*.

[14] Ooi K., Khoo P., Vaclavik V. (2019). The potential effects of the statins on the incidence and progression of age-related macular degeneration. *Clinical and Experimental Ophthalmology*.

[15] Al Moujahed A., Ludwig C. A., Davila J., Vail D., Callaway N. F., Moshfeghi D. (2020). Progression of dry to wet age-related macular degeneration in patients receiving intensive statin treatment. *Investigative Ophthalmology & Visual Science*.

[16] Tzotzas T., Apostolopoulou D., Memi E., Efthymiou H., Krassas G. E. (2006). Th-P16:341 Simvastatin reduces the progression of the early stages (Drusen) of age-related macular degeneration: a pilot study. *Atherosclerosis Supplements*.

[17] McCarty C. A., Mukesh B. N., Guymer R. H., Baird P. N., Taylor H. R. (2001). Cholesterol‐lowering medications reduce the risk of age‐related maculopathy progression. *Medical Journal of Australia*.

[18] Klein R., Klein B. (2004). Do statins prevent age-related macular degeneration?. *American Journal of Ophthalmology*.

[19] van Leeuwen R., Vingerling J. R., Hofman A., de Jong P. T., Stricker B. H. (2003). Cholesterol lowering drugs and risk of age related maculopathy: prospective cohort study with cumulative exposure measurement. *BMJ*.

[20] Klein R., Klein B. E., Tomany S. C., Danforth L. G., Cruickshanks K. J. (2003). Relation of statin use to the 5-year incidence and progression of age-related maculopathy. *Archives of Ophthalmology*.

[21] Klein R., Klein B. E., Knudtson M. D. (2007). Subclinical atherosclerotic cardiovascular disease and early age-related macular degeneration in a multiracial cohort. *Archives of Ophthalmology*.

[22] Tan J. S. L., Mitchell P., Rochtchina E., Wang J. J. (2007). Statins and the long-term risk of incident age-related macular degeneration: the Blue Mountains Eye Study. *American Journal of Ophthalmology*.

[23] Maguire M. G., Ying G.-S., McCannel C. A., Liu C., Dai Y. (2009). Statin use and the incidence of advanced age-related macular degeneration in the complications of age-related macular degeneration prevention trial. *Ophthalmology*.

[24] Smeeth L., Douglas I., Hall A. J., Hubbard R., Evans S. (2009). Effect of statins on a wide range of health outcomes: a cohort study validated by comparison with randomized trials. *British Journal of Clinical Pharmacology*.

[25] Shalev V., Sror M., Goldshtein I., Kokia E., Chodick G. (2011). Statin use and the risk of age related macular degeneration in a large health organization in Israel. *Ophthalmic Epidemiology*.

[26] Vanderbeek B. L., Zacks D. N., Talwar N., Nan B., Stein J. D. (2013). Role of statins in the development and progression of age-related macular degeneration. *Retina*.

[27] Cougnard-Grégoire A., Delyfer M. N., Korobelnik J. F. (2014). Elevated high-density lipoprotein cholesterol and age-related macular degeneration: the Alienor study. *PLoS One*.

[28] Al-Holou S. N., Tucker W. R., Agrón E. (2015). The association of statin use with age-related macular degeneration progression. *Ophthalmology*.

[29] Cruickshanks K. J., Nondahl D. M., Johnson L. J. (2017). Generational differences in the 5-year incidence of age-related macular degeneration. *JAMA Ophthalmology*.

[30] Ludwig C. A., Vail D., Rajeshuni N. A. (2021). Statins and the progression of age-related macular degeneration in the United States. *PLoS One*.

[31] McGwin G., Owsley C., Curcio C. A., Crain R. J. (2003). The association between statin use and age related maculopathy. *British Journal of Ophthalmology*.

[32] Smeeth L., Cook C., Chakravarthy U., Hubbard R., Fletcher A. E. (2005). A case control study of age related macular degeneration and use of statins. *British Journal of Ophthalmology*.

[33] McGwin G., Modjarrad K., Hall T. A., Xie A., Owsley C. (2006). 3-hydroxy-3-methylglutaryl coenzyme a reductase inhibitors and the presence of age-related macular degeneration in the Cardiovascular Health Study. *Archives of Ophthalmology*.

[34] Etminan M., Brophy J., Maberley D. (2008). Use of statins and angiotensin converting enzyme inhibitors (ACE-Is) and the risk of age-related macular degeneration: nested case-control study. *Current Drug Safety*.

[35] Kaiserman N., Vinker S., Kaiserman I. (2009). Statins do not decrease the risk for wet age-related macular degeneration. *Current Eye Research*.

[36] Fong D. S., Contreras R. (2010). Recent statin use and 1-year incidence of exudative age-related macular degeneration. *American Journal of Ophthalmology*.

[37] Lee H., Jeon H.-L., Park S. J., Shin J.-Y. (2019). Effect of statins, metformin, angiotensin-converting enzyme inhibitors, and angiotensin II receptor blockers on age-related macular degeneration. *Yonsei Medical Journal*.

[38] Cackett P., Wong T. Y., Aung T. (2008). Smoking, cardiovascular risk factors, and age-related macular degeneration in Asians: the Singapore Malay Eye Study. *American Journal of Ophthalmology*.

[39] Barbosa D. T. Q., Mendes T. S., Cíntron-Colon H. R. (2014). Age-related macular degeneration and protective effect of HMG Co-A reductase inhibitors (statins): results from the National Health and Nutrition Examination Survey 2005-2008. *Eye*.

[40] Rajeshuni N., Ludwig C. A., Moshfeghi D. M. (2019). The effect of statin exposure on choroidal neovascularization in nonexudative age-related macular degeneration patients. *Eye*.

[41] Tsao S. W., Fong D. S. (2013). Do statins have a role in the prevention of age-related macular degeneration?. *Drugs & Aging*.

[42] Snow K. K., Seddon J. M. (1999). Do age-related macular degeneration and cardiovascular disease share common antecedents?. *Ophthalmic Epidemiology*.

